# Rapid disease progression on immune checkpoint inhibitors in young patients with stage IV melanoma

**DOI:** 10.3389/fmed.2023.1117816

**Published:** 2023-01-23

**Authors:** Devayani Machiraju, Sarah Schäfer, Philip Beckhove, Jasmin Roth, Carsten Schulz, Jessica C. Hassel

**Affiliations:** ^1^Department of Dermatology, National Center for Tumor Diseases, University Hospital Heidelberg, Heidelberg, Germany; ^2^Regensburg Center for Interventional Immunology, University Hospital Regensburg, Regensburg, Germany

**Keywords:** melanoma, young adults, memory T cells, soluble immune checkpoint proteins, age, immune checkpoint inhibitors (ICI)

## Abstract

**Background:**

Immune checkpoint inhibitors (ICIs) are the standard of care for metastatic cutaneous melanoma (mCM) patients, but their efficacy in young adults aged less than 40 years remains unclear.

**Materials and methods:**

We retrospectively analyzed 303 stage IV melanoma patients of different ages treated with nivolumab, pembrolizumab, or ipilimumab plus nivolumab combination therapy. Clinical data and blood values such as LDH, CRP, and absolute immune cell counts were retrieved from the medical records. Pre-treatment serum concentrations of soluble immune checkpoint proteins were measured using ELISA. In addition, information on frequencies of various T cell subsets in the peripheral blood was collected from a previously reported study (ELEKTRA). Patient characteristics and clinical information was correlated with PFS and OS using univariate and multivariate cox regression analysis.

**Results:**

Of 303 patients, 33 (11%) were ≤ 40 years old. The older patients had a median age of 64 (95% CI: 61–66). Concerning prognostic parameters, there was no difference between the age groups, e.g., in gender, LDH, or the existence of brain or liver metastases. Patients aged ≤ 40 years [*p* = 0.014; HR: 1.6 (95% CI: 1.1–2.4)], presence of liver metastases [*p* = 0.016; HR: 1.4 (95% CI: 1.0–1.9)], line of ICI treatment [*p* = 0.009; HR: 1.4 (1.0–1.9)], elevated LDH [*p* = 0.076; HR: 1.3 (95% CI: 0.97–1.8)], and brain metastasis [*p* = 0.080; HR: 1.3 (95% CI: 0.97–1.7)], were associated with shorter PFS in univariate analysis. Multivariate analysis revealed that the patient’s age (≤ 40 years) remains a high-risk factor upon adjusting for all potential confounders [*p* = 0.067; HR: 1.5 (95% CI: 0.97–2.3)]. Blood parameters revealed that patients ≤ 40 years have relatively higher frequencies of activated CD4 T cells (CD4 + Ki67 + CD4 + ICOS +) in the blood, and significantly lower number of basophils and CD45RA- memory T cells, compared to patients above 40 years (*p* < 0.05). In addition, patients ≤ 40 years experiencing disease progression within 6 months of ICI treatment had increased concentrations of sPDL1 (*p* = 0.05) and sTIM3 (*p* = 0.054) at baseline.

**Conclusion:**

Young patients with stage IV melanoma may experience shorter progression-free survival upon ICI treatment compared to patients above 40 years and are characterized by fewer basophils and memory T cells in the blood.

## 1. Introduction

Melanoma is one of the most frequent cancers observed in young adults aged less than 40 years (1, 2). Although most young patients are diagnosed early, the prognosis gets bad if the melanoma has already metastasized to distant organs, with a 5-years survival rate of only 12–20% (3–6). Immunotherapy with immune checkpoint inhibitors (ICIs) such as anti-programmed cell death protein 1 (anti-PD1) alone or in combination with anti-cytotoxic T-lymphocyte associated protein 4 (anti-CTLA4) has shown remarkable survival benefits in metastatic cutaneous melanoma (mCM) patients and is currently the standard of care (7–9). The ICIs activate the exhausted tumor-specific T cells and thus revive anti-tumor activity (10–12). However, the ICI treatment recommendation for young adults is based on clinical trials that are predominantly carried out in patients above 40 years.

Of note, cancer, including melanoma and immune responses, are highly related to biological age. In general, immune responses weaken with age, whereas the risk of cancer increases. Besides environmental factors, one explanation is that a highly active and tightly regulated immune system at a younger age makes it difficult for tumors to spread, whereas as age increases, the immune system enters the immunosenescence stage, favoring tumor development (13, 14). Therefore, it was initially expected that younger patients might experience potential benefits from ICIs; however, recent reports reveal a different picture: mCM patients below 60–65 years are more likely to not respond to anti-PD1 therapy compared to patients above the age, suggesting an influence of patient’s age on ICI efficacy (15). However, the average age cut-off commonly used in these studies is around 60 years; therefore, the efficacy of ICIs in young adult patients remains unclear. Hence, in this study, we investigated the ICI efficacy in young adult patients with stage IV melanoma and analyzed several potential biomarkers in the peripheral blood.

## 2. Materials and methods

### 2.1. Patients

Stage IV cutaneous melanoma patients who received ICI treatment with pembrolizumab, nivolumab, or ipilimumab plus nivolumab between May 2013 and August 2021 were identified from routine patient documentation at the Section of Dermatooncology, NCT Heidelberg. Patient characteristics and analytical information on clinical blood parameters at baseline were retrospectively extracted from the medical records. Laboratory values such as LDH, CRP, absolute immune cell counts (per nl), including neutrophils, monocytes, lymphocytes, eosinophils, and basophils were included if obtained at a maximum of 8 weeks before immunotherapy initiation. The closest to the treatment start was chosen if more than one value was available. Thirty-eight patients who experienced disease progression after only one treatment cycle or died within a month of treatment initiation were excluded from the analysis (flow chart diagram, Supplementary Figure 1). In addition, young patients who agreed to the biobanking of serum samples were identified for soluble protein analysis. The Ethical Committee approved the retrospective analysis of patient data and use of biobanking material of the Medical Faculty of Heidelberg (S-454/2015; S-207/2005).

### 2.2. Serum collection

Pre-treatment peripheral blood samples from young patients were collected using S-Monovette^®^ Serum-Gel (SARSTEDT) tubes and processed according to the standard NCT biobank protocols. The blood samples were centrifuged at 2,500 × *g* for 10 min for serum separation, divided into 200–300 uL aliquots, and stored at −80°C until further analysis.

### 2.3. ELISA

Serum concentrations of soluble immune checkpoint proteins were measured using commercially available ELISA kits according to the manufacturer’s instructions [PD1 kit (#LS-F470-1, LS Bio), PDL1 kit (#ab214565, Abcam), TIM3 kit (#ab231932, Abcam), LAG3 kit (#ab193707, Abcam)]. The data was analyzed using 4PL analysis.

### 2.4. Peripheral T cell frequencies

Flow cytometric data of peripheral T-cell frequencies were collected from a previously reported study (ELEKTRA) (16). The information on frequencies of peripheral T cell subsets in patients with stage IV skin cancer at baseline is available from 71 patients from the ELEKTRA study. Among these patients, 66% overlap with the current study cohort. T cell subsets such as: CD3 + CD4 + ; CD3 + CD4-; CD45RA-; Ki67 +; CD4 + Ki67 + CD45RA-CD4 + ; CD4 + CD127 +; CD25 + CD127-; CD25 + Foxp3 +; CD25 + Foxp3 +; CD25 + CD127-; Ki67 + Foxp3 +; CD4 + CD45RA-; Ki67-CD45RA +; Ki67 + CD45RA +; Ki67 + CD45RA-; Ki67-CD45RA-; Tregs; CD45RA-Ki67 + Tregs; CD25 + LAP +; CD25 + ICOS +; LAP + ICOS +; CD4 + ICOS +; LAP + Tregs; and ICOS + Tregs, analyzed before the initiation of treatment in ELEKTRA study (visit 1) were examined for age differences.

### 2.5. Statistics

Baseline patient characteristics and the clinical variables between the age groups were compared using Mann–Whitney U (MWU) or Chi-square test. Survival curves were calculated by the Kaplan-Meier and compared using the log-rank test. Univariable and multivariable analyses were conducted with Cox regression models. Variables with a *p*-value < 0.1 on univariable analysis were considered for multivariable analysis. Progression-free survival was defined as the time from the start of immunotherapy until disease progression; not progressing patients were censored at the last contact date. Overall survival (OS) was defined as the time from the start of immunotherapy until death, and patients who were still alive were censored at the date of the last contact. All statistical analyses were performed using SPSS version 29 (IBM), and the graphs for data visualization were created using GraphPad Prism version 8 (GraphPad Software, Inc., La Jolla, CA, USA). The bars and lines in the column graphs represent median values and 95% CI. All reported *p*-values are two-sided; *p* < 0.05 indicated a statistically significant difference.

## 3. Results

### 3.1. Patient characteristics

Three hundred and three patients with stage IV cutaneous melanoma receiving ICI treatment were included in the study. Among them, thirty-three patients were aged less than or equal to 40 years at the time of treatment initiation (young patients), and two-hundred and seventy patients were aged above 40 years [middle-aged (41–65 years) or elderly (> 65 years)]. Baseline characteristics and clinical outcomes according to the age groups are described in Table 1. There were no significant differences in patient characteristics, e.g., gender, BRAF mutation status, or serum lactate dehydrogenase (LDH) levels, between the age groups. Concerning treatment, young patients more often received combined immunotherapy with ipilimumab plus nivolumab than anti-PD1 monotherapy. Despite this, the median PFS was only 3 (95% CI: 2–6) months in young patients and 6 (95% CI: 5–8) months in patients above 40. Median OS was 16 (95% CI: 9–33) months in young patients and 22 (95% CI: 18–27) months in patients above 40.

**TABLE 1:** Patient characteristics and clinical outcomes based on age.

	≤ 40 patients (33)	> 40 patients (270)	*P*-value
**Age (years)**			
Median (95% CI)	35 (32–37)	64 (61–66)	
	**n (%)**	**n (%)**	
Gender			0.260
Male	16 (48)	163 (60)	
Female	17 (52)	107 (40)	
Braf			0.361
Mutation	18 (55)	120 (44)	
Wildtype	15 (45)	142 (53)	
Missing		8 (3)	
LDH			0.252
Normal	16 (49)	172 (64)	
Elevated	10 (30)	64 (24)	
Missing	7 (21)	34 (12)	
CRP			0.837
Normal	14 (43)	121 (45)	
Elevated	12 (36)	121 (45)	
Missing	7 (21)	28 (10)	
Brain metastases			1.00
Yes	16 (48)	114 (42)	
No	17 (52)	156 (58)	
Liver metastases			0.675
Yes	10 (30)	79 (29)	
No	23 (70)	191 (71)	
Type of ICI			**0.039**
Pembro/Nivo	13 (39)	161 (60)	
IpiNivo	20 (61)	109 (40)	
Line of ICI treatment			0.199
First line	15 (45)	155 (57)	
Others	18 (55)	115 (43)	
irAEs			0.448
Yes	15 (45)	101 (37)	
No	18 (55)	169 (63)	
PFS (Months)			**0.048**
Median (95% CI)	3 (2–6)	6 (5–8)	
OS (Months)			0.230
Median (95% CI)	16 (9–33)	22 (18–27)	

Bold values denote statistical significance at the *p* < 0.05 level.

### 3.2. Rapid disease progression in young patients with age less than or equal to 40 years

**FIGURE 1:**
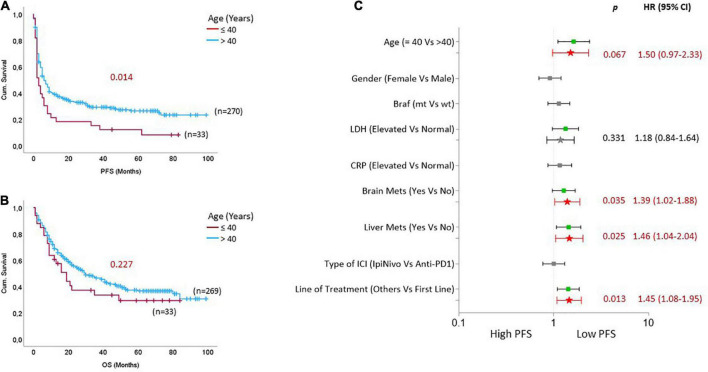
Rapid disease progression on immune checkpoint inhibitors (ICIs) in young patients with stage IV cutaneous melanoma. Kaplan Meier curves based on age groups for **(A)** progression-free survival and **(B)** overall survival. **(C)** The forest plot indicates univariate (box symbol) and multivariate regression (star symbol) analysis for PFS of ICI-treated mCM patients. The green color in the plot indicates that this clinical feature is a high-risk factor for PFS in univariate analysis, and the bright red indicates a high-risk factor in the multivariate model. *P*-values from the multivariate analysis are presented on the graph.

We compared the difference in PFS and OS between young patients and patients above 40 years (Figure 1) and observed that young patients revealed a significantly shorter PFS [*p* = 0.014; HR: 1.6 (95% CI: 1.1–2.4)] and likely shorter OS [*p* = 0.227; HR: 1.3 (95% CI: 0.84–2.0)] compared to patients above 40 years (Figures 1A, B). Along with the young age of patients, the presence of liver metastases [*p* = 0.016; HR: 1.4 (95% CI: 1.0–1.9)], line of ICI treatment [*p* = 0.009; HR: 1.4 (1.0–1.9)], elevated LDH [*p* = 0.076; HR: 1.3 (95% CI: 0.97–1.8)], and the existence of brain metastasis [*p* = 0.080; HR: 1.3 (95% CI: 0.97–1.7)], were associated with shorter PFS (Figure 1C). In addition, the young age of patients remained a trending risk factor for shorter PFS [*p* = 0.067; HR: 1.5 (95% CI: 0.97–2.3)] when adjusted for all potential risk factors (Figure 1C). Younger patients consistently experienced shorter PFS, irrespective of the type of ICI they received. Interestingly, the differences in PFS were wider between younger patients and patients above 80 (Supplementary Figure 2A). However, the patients above 80 years experienced the least overall survival compared to younger patients (Supplementary Figure 2B). In addition, when stratified by the line of ICI treatment, we observed that younger patients experienced worse survival, especially when the ICI treatment was not the first line of treatment (Supplementary Figures 3A, B).

### 3.3. Differences in circulating immune cells

**FIGURE 2:**
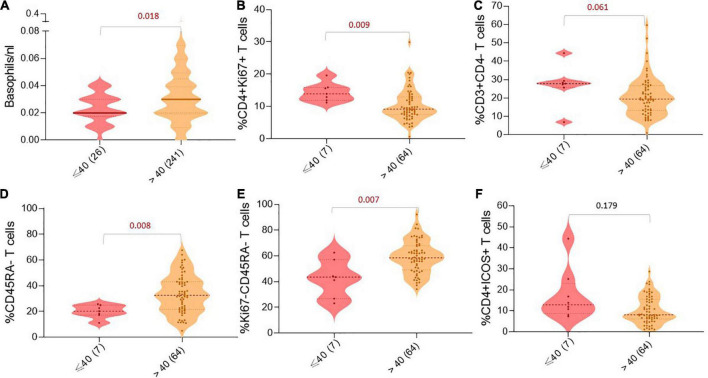
Age-dependent differences in peripheral immune cell populations. Violin charts showing the number/frequency of immune cells in the patient’s blood based on age groups: **(A)** Basophils/nl, **(B)** %CD4 + Ki67 + T cells, **(C)** %CD3 + CD4-T cells, **(D)** %CD45RA-T cells, **(E)** %Ki67-CD45RA-T cells, and **(F)** %CD4 + ICOS + T cells. For all charts, the thick horizontal line and the thin dotted lines represent the 95% CI, respectively. The width of the curved shape indicates the proportion of patients in the group. *P*-values are presented above the respective immune cell subset.

Next, we tried to understand if there are any differences in the absolute immune cell counts or the frequencies of immune cell subsets between the two age groups [young age (≤ 40) versus middle age and elderly patients (> 40)]. We observed that young patients had fewer basophils in the blood than patients above 40 years (*p* < 0.05; Figure 2A). Furthermore, analyzing peripheral immune cell subsets demonstrated that although young patients have increased frequencies of CD4 + Ki67 + and CD3 + CD4- (CD8) T cells, they significantly had lower frequencies of CD45RA-, Ki67-CD45RA-T cells compared to middle-aged and elderly patients (*p* < 0.05; Figures 2B–E). In addition, we also observed a trend of higher frequencies of CD4 + ICOS + T cells in the peripheral blood of young patients at baseline compared to patients above 40 years (*p* = 0.179; Figure 2F), and the difference was more prominent between young patients and patients above 65 years (*p* = 0.043).

### 3.4. High sPDL1 and sTIM3 in rapidly progressing young patients

**FIGURE 3:**
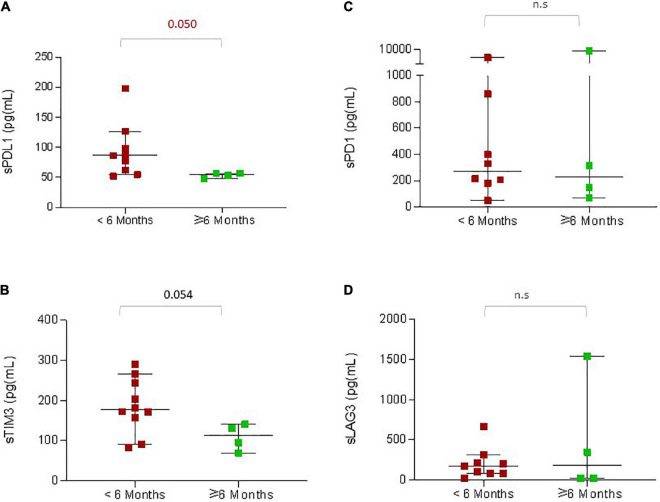
sICPs in younger patients associated with reduced PFS. The graphs indicate pre-treatment concentrations of the soluble immune checkpoint proteins according to the PFS: **(A)** sPDL1, **(B)** sTIM3, **(C)** sPD1, **(D)** sLAG3. The red dots indicate the concentrations of proteins in patients with PFS less than 6 months, and the green dots indicate the concentrations of proteins in patients with PFS greater than 6 months. The *P*-values are presented above the respective bars.

In order to understand the factors associated with rapid disease progression in younger patients, we first compared all the clinical characteristics between patients with a PFS of less than 6 months versus a PFS with more than 6 months (Supplementary Table 1). We noticed that increased LDH, liver metastasis, and ICI not first line were associated with a shorter PFS of less than 6 months (*p* ≤ 0.05). We then analyzed pre-treatment serum concentrations of soluble immune checkpoints (Figures 3A–D). Among the 33 young patients in this study, serum samples were available from 15 patients for analysis. We observed that within this small sub-group, patients who experienced rapid disease progression within 6 months had increased concentrations of sPDL1 (*p* = 0.050) and sTIM3 (*p* = 0.054; Figures 3A, B) at baseline.

## 4. Discussion

In this study, we investigated the survival benefits of ICIs in young adult patients with stage IV melanoma. We observed a strong tendency toward shorter PFS and OS in younger patients compared to patients above 40 years. Adjusting for prognostic features such as brain and liver metastases, systemic line of treatment, and tumor burden revealed that young age is consistently associated with worse progression-free survival upon treatment. Interestingly, this relationship is more evident in patients when the ICI treatment is not the first-line systemic treatment. We also found that young patients had fewer basophils and memory T cells in the circulating blood compared to the middle age and elderly patient population. Besides, increased LDH, liver metastasis, line of ICI treatment, and increased concentrations of sPDL1 (and sTIM3) at baseline were significantly associated with worse outcomes in younger patients. These findings are important as data on outcomes with ICIs in younger patients have been lacking.

Several studies have previously reported age-related differences in the outcomes of melanoma patients treated with anti-PD1 antibodies; however, the average age cut-off among these studies was around 60 years (15). For example, in a retrospective analysis, melanoma patients below the age of 62 experienced a lower disease control rate upon pembrolizumab treatment than patients above 62 (17). Another study observed a clear decrease in OS of melanoma patients younger than 60 upon ICIs (18). Similarly, a meta-analysis including pan-cancer patients further demonstrated that melanoma patients aged 50 years or younger receiving ICIs had shorter OS than older patients. The pooled OS HR for younger patients was 0.74 compared to 0.5 for older patients (19). Although the age cut-offs vary across these studies, they commonly suggest that ICI efficacy may vary with age, and especially patients with lower age are less likely to benefit from ICIs, in line with our findings in this study. Collectively these data suggest that the tumors developing in younger people may have evolved with strong immune editing mechanisms or are able to undergo rapid reprogramming of the signaling pathways upon ICIs. Meanwhile, a Dutch study recently observed no differences in the outcomes of ICI treatment when given as a first-line treatment in adolescents and young adults aged 15–39 years compared to patients over 40 (20). Although there is a variation in the selection criteria between the two studies making it hard to compare, we also observed that PFS variation among different age groups was highly significant when ICIs were not given as a first-line treatment. Therefore, it is crucial to investigate the ICI efficacy in young adult patients using larger cohorts and to evolve strategies to maximize the clinical benefits in this population.

Among the population analyzed for immune cell subsets in the peripheral blood in this study, the age-related differences were majorly observed in circulating basophils and memory T cells, as both tend to be reduced in younger patients. Basophils represent < 1% of leucocytes in the blood; despite being a very small proportion of circulating immune cells, they are potent immune effector cells. The role of basophils in melanoma has been previously demonstrated in the Treg-depleted melanoma mouse model, which was associated with the infiltration of basophils and CD8 + T cells, leading to an anti-tumor immune attack. Basophils promote CD8 + infiltration into the tumors, and depletion of basophils prevents melanoma rejection by the immune system (21). Therefore, fewer basophils in young patients observed in our study may contribute to their outcomes with ICIs. In addition, as young patients are more likely subjected to cancer-related anxiety and depression compared to elderly patients, the low numbers of basophils in young patients may also reflect the stressful experience, which can further compromise ICI efficacy in this population (22, 23). Besides, one of the most important consequences of adaptive immune responses to melanomas is establishing a state of immunological memory as tumor-specific memory T cells exert anti-tumor effects after short-term stimulation (24). The CD45RA is expressed on naive T cells, which respond poorly to recall antigens, and when activated by a foreign or tumor antigen, they lose expression of the CD45RA (25). Increased frequencies of CD45RA-memory T cells were previously shown to be associated with better responses to ICIs in melanoma patients (16). Therefore, the lower frequencies of these T cells in the blood may suggest the absence of tumor-specific T cell memory in younger patients required for a successful ICI treatment. Interestingly, basophils are also known to augment effector functions of memory CD4+ T cells (26). Hence, lower frequencies of both basophils and memory T cells observed in young patients in our study may partly contribute to the results we observed. However, it has to be kept in mind that the development of immune memory is highly age-dependent as nearly all T cells are CD45RA +, typical of naive T cells at birth, and as we get older, we develop an expanding repertoire of memory T cells shaped by exposure to various microbes, food, and tumor antigens over the lifespan (27).

Although the study tried to investigate ICI efficacy in younger patients, several limitations must be noted. Firstly, this study is single-centered and retrospectively analyzed; therefore might have been subjected to a potential selection bias. Although we added all patients enrolled at our institution until 2021, over a nearly 9-years period, our sample size is relatively small, particularly at the age < 40; therefore, our finding warrants confirmation in additional larger cohorts in the future. Finally, despite adjusting for potential factors influencing the results in our study using a multivariate model, unmeasured confounders are possible. Further studies are required to clarify and enhance the presence of tumor-specific immune memory in younger patients. In addition, it is important to note that the findings on patients’ age-wise differences in peripheral immune cells and the clinical significance of soluble immune checkpoint proteins in young patients reported in our study are based on small cohorts. Therefore further studies are strictly required to assess the generalizability of these findings.

In conclusion, young patients with stage IV melanoma may experience rapid disease progression during ICI treatment, especially when not given as first-line systemic treatment. Therefore, managing young adult patients with mCM should be tailored based on individual evaluation. As mCM rarely affects younger adults, our work underscores the importance of including these patients in clinical trials to understand better the efficacy and safety of novel cancer therapies in populations representative of those seen in routine. In addition, methods to enhance tumor-specific immune memory should be further investigated in younger patients.

## Data availability statement

The original contributions presented in this study are included in this article/Supplementary material, further inquiries can be directed to the corresponding author.

## Ethics statement

The studies involving human participants were reviewed and approved by Medical Faculty of Heidelberg (S-454/2015 and S-207/2005). The patients/participants provided their written informed consent to participate in this study.

## Author contributions

DM, SS, and JH conceived and designed the overall study. CS and JR worked on data acquisition. DM and SS worked on data analysis, interpretation, and prepared the original manuscript. JH supervised and reviewed the manuscript. All authors have read and approved the final version of the manuscript.
