# Supplementary material for: Rapid disease progression on immune checkpoint inhibitors in young patients with stage IV melanoma
Source: Front Med (Lausanne). 2023 Jan 23;10:1117816. doi: 10.3389/fmed.2023.1117816 (PMC9899839; doi:10.3389/fmed.2023.1117816)
Supplement: Supplementary file 1 [file Presentation_1.PPTX]

## Slide 1
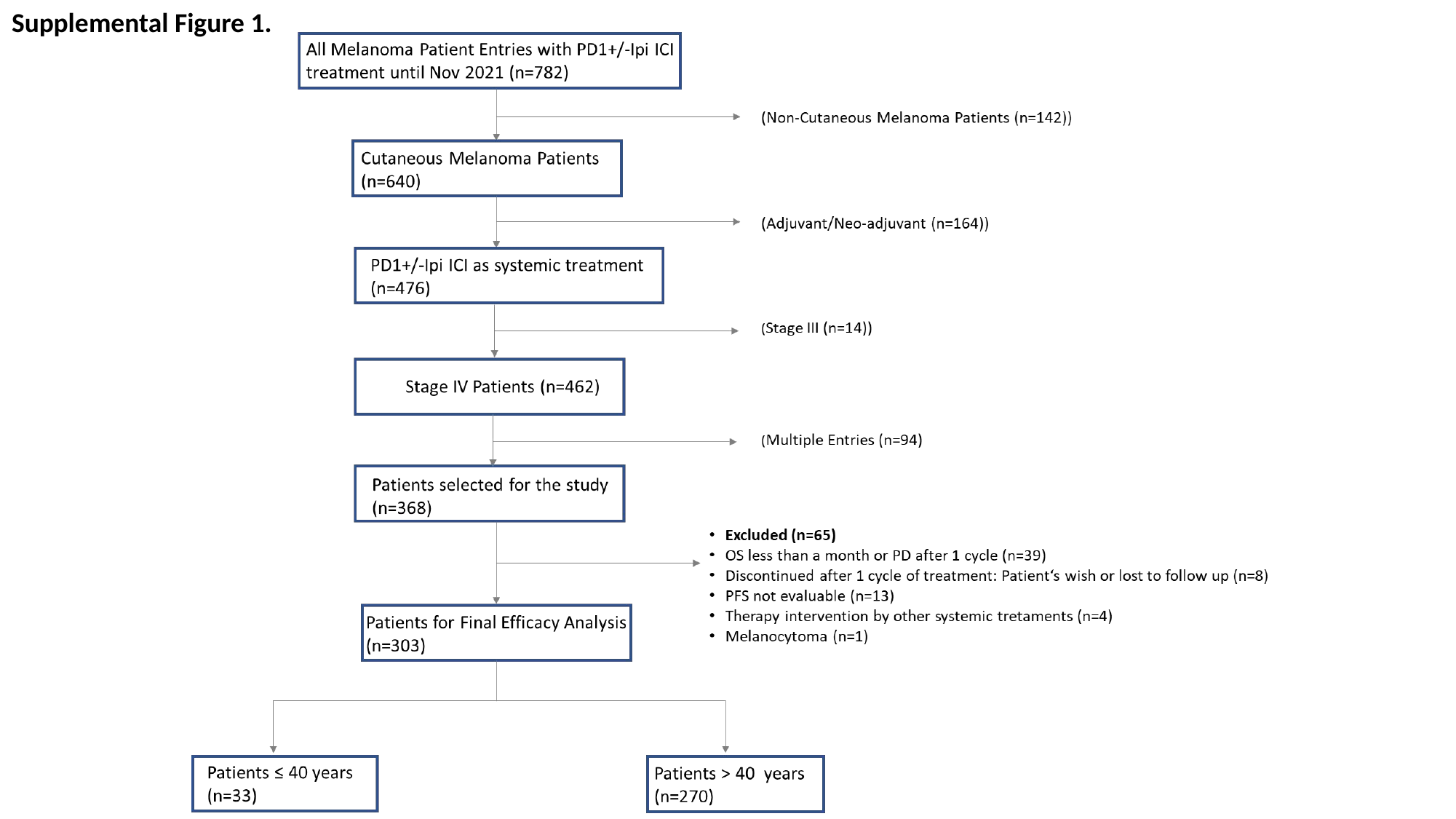

Supplemental Figure 1.

## Slide 2
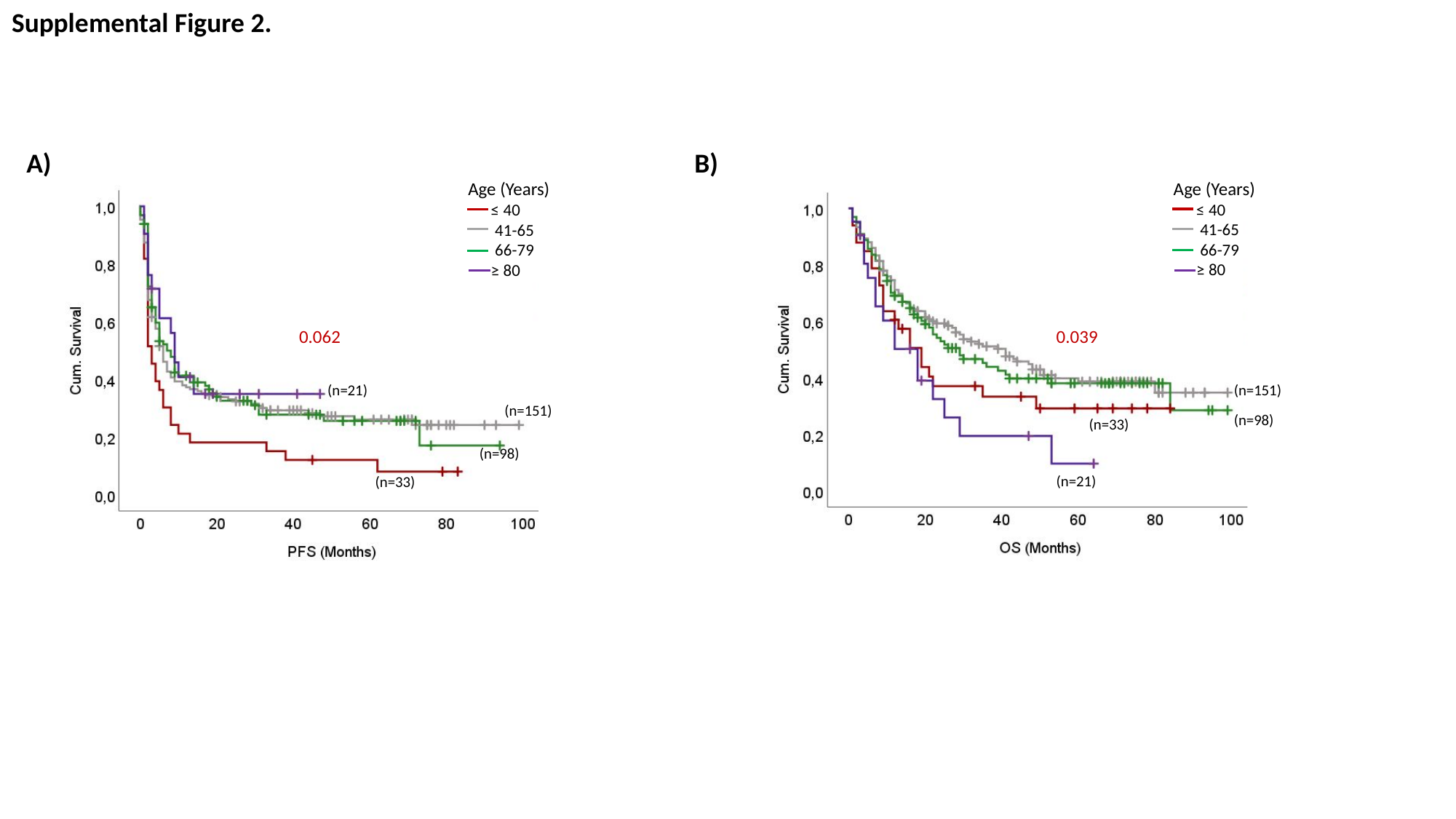

Supplemental Figure 2.
A)
B)
Age (Years)
 ≤ 40
 41-65
 66-79
 ≥ 80
Age (Years)
 ≤ 40
 41-65
 66-79
 ≥ 80
0.062
0.039
(n=151)
(n=21)
(n=151)
(n=98)
(n=33)
(n=98)
(n=21)
(n=33)

## Slide 3
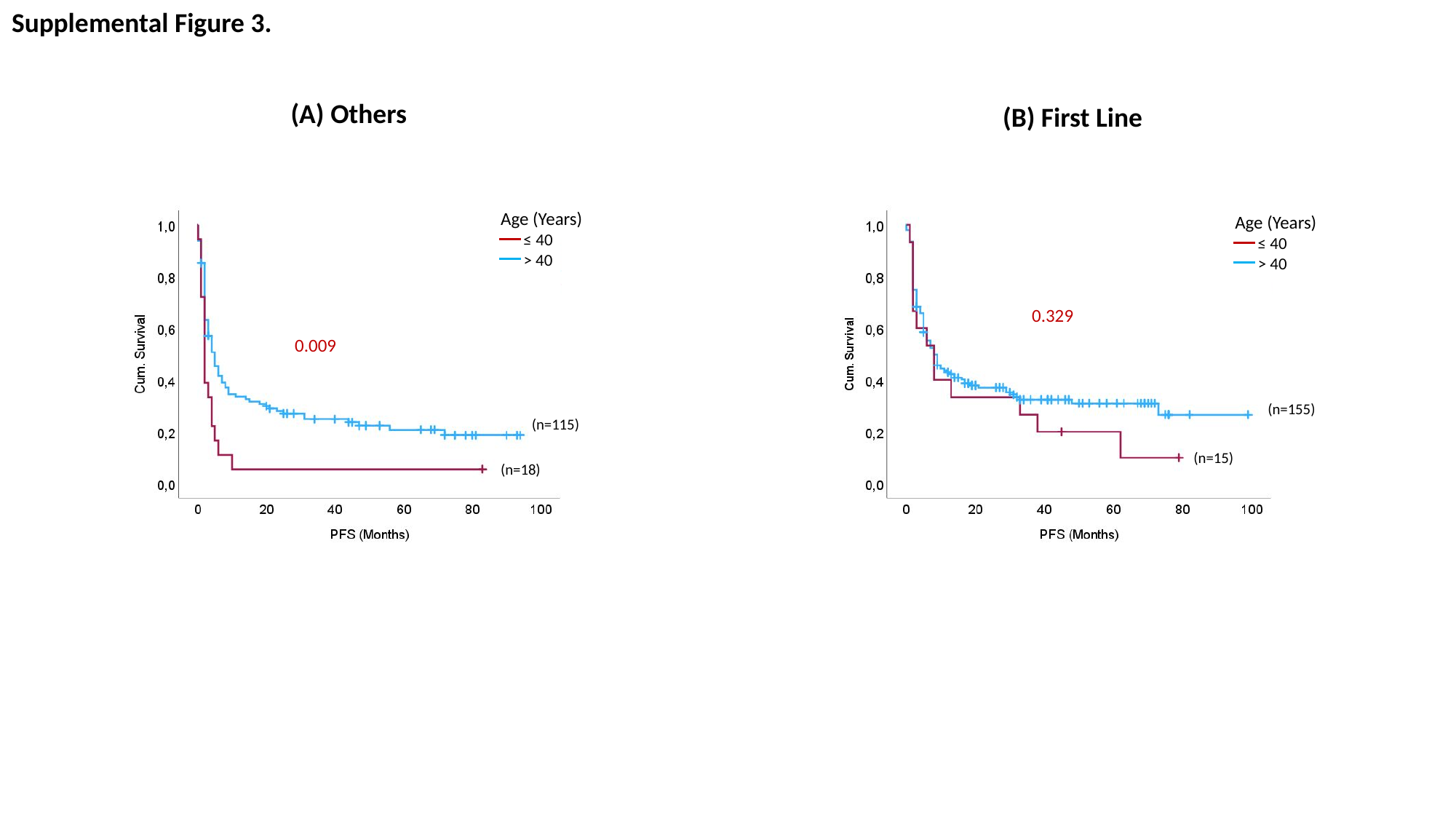

Supplemental Figure 3.
(A) Others
 (B) First Line
Age (Years)
 ≤ 40
 > 40
Age (Years)
 ≤ 40
 > 40
0.329
0.009
(n=155)
(n=115)
(n=15)
(n=18)
