# Supplementary material for: Rapid disease progression on immune checkpoint inhibitors in young patients with stage IV melanoma
Source: Front Med (Lausanne). 2023 Jan 23;10:1117816. doi: 10.3389/fmed.2023.1117816 (PMC9899839; doi:10.3389/fmed.2023.1117816)
Supplement: Supplementary file 2 [file Table_1.DOCX]

| **Supplementary Table 1:** Clinical Characteristics of Young Patients based on ICI outcome | | | | |
| --- | --- | --- | --- | --- |
|  |  | **< 6 Months (21)** | **> 6 Months (12)** | ***p* value** |
| **Age (Years)** |  |  |  |  |
| Median (Range) |  | 35 (22-40) | 35 (20-40) |  |
|  |  |  |  |  |
| **Gender** |  |  |  |  |
| Male |  | 10 (48) | 6 (50) | 1.0 |
| Female |  | 11 (52) | 6 (50) |  |
|  |  |  |  |  |
| **Braf** |  |  |  |  |
| mutation |  | 11 (52) | 7 (58) | 1.0 |
| wildtype |  | 10 (48) | 5 (42) |  |
| Missing |  | 0 |  |  |
|  |  |  |  |  |
| **LDH** |  |  |  | **0.05** |
| Normal |  | 6 (29) | 10 (83) |  |
| Elevated |  | 8 (38) | 2 (17) |  |
| Missing |  | 7 (33) |  |  |
|  |  |  |  |  |
| **CRP** |  |  |  | 0.26 |
| Normal |  | 6 (29) | 8 (67) |  |
| Elevated |  | 8 (38) | 4 (33) |  |
| Missing |  | 7 (33) |  |  |
|  |  |  |  |  |
| **Brain Metastases** |  |  |  | 0.13 |
| Yes |  | 10 (48) | 2 (17) |  |
| No |  | 11 (52) | 10 (83) |  |
|  |  |  |  |  |
| **Liver Metastases** |  |  |  | **0.05** |
| Yes |  | 9 (43) | 1 (8) |  |
| No |  | 12 (57) | 11 (92) |  |
|  |  |  |  |  |
| **Type of ICI** |  |  |  | 1.0 |
| Pembro/Nivo |  | 8 (38) | 5 (42) |  |
| IpiNivo |  | 13 (62) | 7 (58) |  |
|  |  |  |  |  |
| **Line of ICI Treatment** |  |  |  | **0.01** |
| First Line |  | 6 (29) | 9 (75) |  |
| Others |  | 15 (71) | 3 (25) |  |
